# Development and validation of a nomogram to predict the risk of renal replacement therapy among acute kidney injury patients in intensive care unit

**DOI:** 10.1007/s10157-023-02383-5

**Published:** 2023-07-27

**Authors:** Jiang-Chen Peng, Yan Wu, Shun-Peng Xing, Ming-Li Zhu, Yuan Gao, Wen Li

**Affiliations:** 1 Department of Critical Care, Ren Ji Hospital, School of Medicine, Shanghai Jiao Tong University, 160 Pujian Road, Shanghai, 200127 China; 2 Department of Critical Care, Shanghai Baoshan Luodian Hospital, 121 Luoxi Road, Baoshan District, Shanghai, 201908 China

**Keywords:** Acute kidney injury, Renal replacement therapy, Nomogram, Intensive care unit, MIMIC-III database

## Abstract

**Background:**

There are no universally accepted indications to initiate renal replacement therapy (RRT) among patients with acute kidney injury (AKI). This study aimed to develop a nomogram to predict the risk of RRT among AKI patients in intensive care unit (ICU).

**Methods:**

In this retrospective cohort study, we extracted AKI patients from Medical Information Mart for Intensive Care III (MIMIC-III) database. Patients were randomly divided into a training cohort (70%) and a validation cohort (30%). Multivariable logistic regression based on Akaike information criterion was used to establish the nomogram. The discrimination and calibration of the nomogram were evaluated by Harrell’s concordance index (*C*-index) and Hosmer–Lemeshow (HL) test. Decision curve analysis (DCA) was performed to evaluate clinical application.

**Results:**

A total of 7413 critically ill patients with AKI were finally enrolled. 514 (6.9%) patients received RRT after ICU admission. 5194 (70%) patients were in the training cohort and 2219 (30%) patients were in the validation cohort. Nine variables, namely, age, hemoglobin, creatinine, blood urea nitrogen and lactate at AKI detection, comorbidity of congestive heart failure, AKI stage, and vasopressor use were included in the nomogram. The predictive model demonstrated satisfying discrimination and calibration with *C*-index of 0.938 (95% CI, 0.927–0.949; HL test,* P* = 0.430) in training set and 0.935 (95% CI, 0.919–0.951; HL test, *P* = 0.392) in validation set. DCA showed a positive net benefit of our nomogram.

**Conclusion:**

The nomogram developed in this study was highly accurate for RRT prediction with potential application value.

**Supplementary Information:**

The online version contains supplementary material available at 10.1007/s10157-023-02383-5.

## Introduction

The incidence of acute kidney injury (AKI) in intensive care unit (ICU) occurs in approximately 50% of critically ill patients [1]. The reason for AKI is often multi-factorial and complicated. If the renal injury cannot be reversed, then renal replacement therapy (RRT) would be considered. The incidence of AKI patients treated with RRT is approximately 5–10% [1, 2]. However, hospital mortality of AKI patients treated with RRT is generally above 50% in ICU [3]. RRT is known to increase the ICU survival rate due to its ability to correct metabolic acidosis by removing lactate unmeasured anions, phosphate, and chloride. Nevertheless, the indication to initiate RRT is still various and controversial [4–6], and there are no universally standard of urea, creatinine, potassium, or pH to guide clinical treatment. What’s more, several factors, both renal and non-renal, have influence on initiating RRT [7]. Therefore, it is important to develop a generally accepted tool for prediction of RRT in AKI patients, which may aid in delivering proper care and optimizing the use of limited resources.

Nomograms are popular predictive tools with the ability to combine potential risk factors, which could be easily applied in clinical practice to stratify patients with high risk and guide clinical decision-making [8]. In this study, we aimed to construct a nomogram based on multivariate logistic regression model for the prediction of RRT among AKI patients in ICU and make internal validation. Medical Information Mart for Intensive Care III (MIMIC-III) database can provide a wealth of clinical data, which could be routinely analyzed. Through examining the AKI epidemiology confined in intensive care unit, we hoped to investigate the likelihood of RRT by nomogram with extensive clinical data ranging from demographics, comorbidities, biochemistries, and treatment therapies.

## Materials and methods

### Data source

We conducted this retrospective study based on MIMIC-III version 1.4 (MIMIC-III v1.4) database. MIMIC-III, a large and public database, contains comprehensive data of more than 50,000 patients admitted to the ICU at Beth Israel Deaconess Medical Center from 2001 to 2012 [9]. Before getting access to the database, one author (PJC) has completed the online training course of the National Institutes of Health and obtained access to the database (record ID: 41046393). The establishment and employment of this database were approved by the Institutional Review Boards of the Massachusetts Institute of Technology and Beth Israel Deaconess Medical Center. All methods were performed in accordance with the relevant guidelines and regulations.

### Study population

Patients meeting criteria for Kidney Disease: Improving Global Outcomes (KDIGO) serum creatinine (sCr) definition were enrolled for this study [10]: an increase in sCr of 26.5 μmol/L within 48 h or an elevation at least 1.5 times the baseline value within 7 days [10]. The baseline sCr level was defined as the minimum sCr measured during the 7 days before ICU admission. When the pre-admission sCr was missing, the first sCr after ICU admission was regarded as the baseline sCr. For patients with multiple hospitalizations, we only used their first hospitalization.

The exclusion criteria included: (i) patients with a diagnosis of end-stage renal disease (ESRD) based on International Classification of Diseases, Ninth Revision, Clinical Modification (ICD-9-CM) code 585; (ii) patients already with RRT at ICU admission; (iii) age < 18 or individual missing data > 10%.

### Data extraction

Data were extracted from MIMIC-III database through Structured Query Language. The following variables were collected, such as age, gender, baseline estimated glomerular filtration rate (eGFR) calculated by Chronic Kidney Disease Epidemiology Collaboration (CKD-EPI) equation [10], serum biochemistry at AKI detection (sCr, blood urea nitrogen (BUN), white blood cell (WBC) count, platelet count, hemoglobin, international normalized ratio (INR), albumin, pH, partial pressure of oxygen (*p*O_2_), partial pressure of carbon dioxide (*p*CO_2_), lactate, anion gap, bicarbonate, serum sodium, serum potassium, serum phosphate, and serum chloride), mean values of vital signs at AKI detection [temperature, mean arterial pressure (MAP), respiratory rate, and heart rate], comorbidities [hypertension, diabetes mellitus (DM), congestive heart failure, myocardial infarction (MI), and liver cirrhosis], clinical severity scales [Sequential Organ Failure Assessment (SOFA) score and Simplified Acute Physiology Score II (SAPS II)], and treatment [mechanical ventilation (MV) and vasopressor use]. Outcomes included hospitality mortality and length of stay (LOS) in ICU. For missing variables, predictive mean matching was used to impute numeric features (Additional file 1: Table S1).

### Statistical analysis

Continuous variables are presented as the mean ± standard deviation (SD) for normal distribution and as the median and interquartile range (IQR) for skewed distribution. Normal distributions were confirmed by Agostino tests. Continuous variables were compared by unpaired Student’s test or Mann–Whitney *U* test. Categorical variables were compared using the *χ*^2^ test or Fisher’s exact test as appropriate.

Univariate logistic regression analysis was performed to explore the potential factors with *P* values < 0.1 in the training cohort. Subsequently, the including variables in univariate were used to establish multivariate logistic regression by the backward step-down process. According to the Akaike information criterion (AIC), the best model should achieve a minimum AIC value. Finally, factors with significance in the multivariate logistic regression analysis were utilized to build a prediction model. In the model, the score of each predictor was calculated based on the coefficients of logistic regression variables and a nomogram was used to visualize the model. The variance inflation factor (VIF) was calculated to detect the potential collinearity between continuous variables. When VIF > 10, collinearity was considered to exist and it will be solved by regularization.

The data of 7413 patients were randomly assigned into two complementary subsets: the training set of 5194 patients (70%) was used to establish the model and the validation set of 2219 patients (30%) was used to validate the analysis. The Harrell’s concordance index (*C*-index) and receiver-operating characteristic (ROC) curve were used to evaluate the discrimination ability of the nomogram. Calibration curves were plotted to assess the calibration of each set and were accompanied with the Hosmer–Lemeshow (HL) goodness-of-fit test [11]. Decision curve analysis (DCA) was performed to assess the clinical usefulness of the nomogram by quantifying the standardized net benefits at different threshold probabilities [12].

All analyses were conducted using R software (version 3.6.3) and two-sided *P* values less than 0.05 were considered statistically significant in each statistical analysis.

## Results

### Baseline characteristics of the included patients

**Fig. 1:**
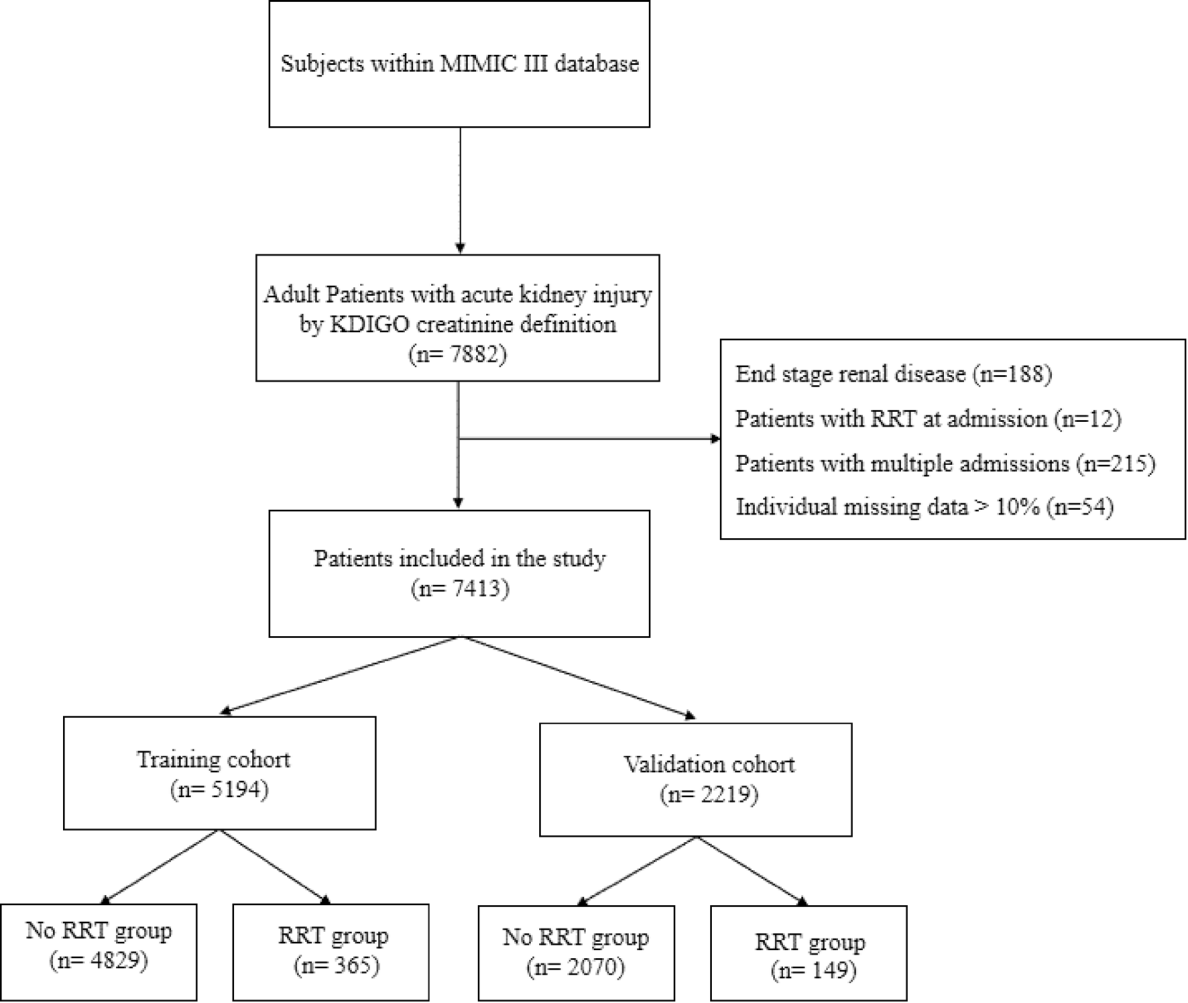
Flowchart of the included patients. *KDIGO* Kidney Disease: Improving Global Outcomes, *MIMIC* Medical Information Mart of Intensive Care, *RRT* renal replacement therapy

A total of 7882 critically ill patients with AKI were obtained in the database. Finally, 7413 eligible patients were enrolled according to the inclusion criteria (Fig. 1). The median age was 70 years. AKI stages at detection were 1 (*n* = 5178, 69.9%), 2 (*n* = 1265, 17.1%), and 3 (*n* = 970, 13.1%), respectively. 514 (6.9%) patients underwent RRT after ICU admission and the median time from AKI diagnosis to RRT initiation was 2.8 days. The training cohort composed of 5194 patients and the validation cohort comprised of 2219 patients. More patients who received RRT (versus none) tended to be younger and had significantly lower baseline eGFR in both training and validation cohorts. As regard to laboratory results at AKI detection, patients who received RRT featured significantly higher levels of INR, sCr, BUN, anion gap, lactate, potassium, and phosphate and lower levels of hemoglobin, platelet count, pH, *p*O_2_, and bicarbonate when compared with patients who did not require RRT (Table 1).

**Table 1:** Baseline characteristics of the study population

	Training cohort			Validation cohort		
	No RRT (*n* = 4829)	RRT (*n* = 365)	*P* value	No RRT (*n* = 2070)	RRT (*n* = 149)	*P* value
Age, years	70 (61, 78)	63 (51, 75)	< 0.001	71 (62, 79)	63 (50, 74)	< 0.001
Male, *n* (%)	2960 (61.3%)	226 (61.9%)	0.814	1273 (61.5%)	102 (68.5%)	0.091
Baseline eGFR (ml/min/1.73 m^2^)	83.8 (54.1, 99.0)	46.4 (29.3, 77.1)	< 0.001	83.8 (55.6, 98.3)	46.5 (28.1, 72.4)	< 0.001
Laboratory tests at AKI detection						
WBC count (10^3^/μL)	13.0 (9.6, 17.2)	14.1 (9.4, 19.8)	0.005	12.8 (9.6, 17.3)	13.8 (9.9, 19.7)	0.068
Hemoglobin (g/dL)	9.2 (7.8, 10.8)	8.8 (7.4, 10.2)	< 0.001	9.2 (7.9, 10.7)	8.8 (7.8, 10.1)	0.021
Platelet count (10^3^/μL)	158.0 (111.0, 220.0)	108.0 (58.0, 190.0)	< 0.001	161.0 (113.0, 224.0)	115.0 (63.0, 187.0)	< 0.001
INR	1.4 (1.2, 1.7)	1.9 (1.4, 2.7)	< 0.001	1.4 (1.2, 1.7)	1.8 (1.4, 2.7)	< 0.001
Serum creatinine (mg/dL)	1.4 (1.1, 2.1)	4.1 (2.8, 5.6)	< 0.001	1.3 (1.1, 2.0)	4.1 (2.8, 5.8)	< 0.001
BUN (mg/dL)	20.0 (15.0, 31.0)	46.0 (27.0, 72.0)	< 0.001	20.0 (15.0, 31.0)	43.0 (27.0, 76.0)	< 0.001
pH	7.39 (7.33, 7.44)	7.33 (7.23, 7.40)	< 0.001	7.39 (7.33, 7.43)	7.34 (7.25, 7.41)	< 0.001
* p*O_2_ (mmHg)	168.0 (94.0, 326.0)	118.0 (79.0, 226.0)	< 0.001	170.0 (95.0, 333.0)	110.0 (79.0, 260.0)	< 0.001
* p*CO_2_ (mmHg)	41.0 (36.0, 46.0)	39.0 (42.0, 46.0)	< 0.001	41.0 (36.0, 47.0)	40.0 (32.0, 47.0)	0.050
Bicarbonate (mg/dL)	22.0 (20.0, 25.0)	17.0 (14.0, 21.0)	< 0.001	23.0 (20.0, 25.0)	18.0 (15.0, 22.0)	< 0.001
Anion gap (mmol/L)	14.0 (12.0, 17.0)	20.0 (17.0, 24.0)	< 0.001	14.0 (12.0, 17.0)	19.0 (16.0, 26.0)	< 0.001
Lactate (mmol/L)	2.6 (1.7, 4.0)	6.8 (3.5, 11.5)	< 0.001	2.5 (1.6, 3.9)	6.8 (3.7, 13.8)	< 0.001
Serum sodium (mmol/L)	136 (134.0, 138.0)	135.0 (131.0, 138.0)	< 0.001	136 (134.0, 138.0)	135.0 (131.0, 138.0)	0.015
Serum potassium (mmol/L)	4.8 (4.3, 5.5)	5.0 (4.4, 6.0)	< 0.001	4.8 (4.3, 5.5)	5.1 (4.5, 5.7)	< 0.001
Serum chloride (mmol/L)	109.0 (104.0, 112.0)	107.0 (101.0, 111.0)	0.033	109.0 (105.0, 112.0)	107.0 (102.0, 112.0)	0.052
Serum phosphate (mg/dL)	3.5 (2.9, 4.1)	4.2 (3.4, 5.7)	< 0.001	3.4 (2.9, 4.1)	4.2 (3.4, 5.4)	< 0.001
Vital signs at AKI detection						
Temperature (℃)	36.8 (36.4, 37.3)	36.6 (36.2, 37.2)	0.184	36.9 (36.5, 37.3)	36.6 (36.1, 37.2)	0.080
MAP (mmHg)	75 (70, 83)	72 (65, 79)	< 0.001	75 (70, 82)	72 (66, 80)	0.004
Respiratory rate (min^−1^)	18 (16, 21)	21 (17, 25)	< 0.001	18 (16, 21)	20 (18, 25)	< 0.001
Heart rate (min^−1^)	84 (76, 94)	89 (77, 103)	< 0.001	84 (76, 94)	93 (80, 106)	< 0.001
Comorbidities, *n* (%)						
Hypertension	2500 (51.8%)	107 (29.3%)	< 0.001	1142 (55.2%)	48 (32.2%)	< 0.001
DM	1581 (32.7%)	108 (29.6%)	0.215	694 (33.5%)	52 (34.9%)	0.732
Congestive heart failure	1715 (35.5%)	146 (40.0%)	0.032	687 (33.2%)	55 (36.9%)	0.046
Myocardial infarction	706 (14.6%)	48 (13.2%)	0.088	328 (15.8%)	17 (17.2%)	0.054
Liver cirrhosis	281 (5.8%)	83 (22.7%)	< 0.001	112 (5.4%)	26 (17.5%)	< 0.001
AKI stage, *n* (%)			< 0.001			< 0.001
Stage 1	3522 (72.9%)	87 (23.8%)		1533 (74.1%)	36 (24.2%)	
Stage 2	809 (16.8%)	66 (18.1%)		363 (17.5%)	27 (8.1%)	
Stage 3	498 (10.3%)	212 (58.1%)		174 (8.4%)	86 (57.7%)	
Treatment, *n* (%)						
MV	6549 (55.6%)	266 (73.7%)	< 0.001	1371 (66.2%)	112 (75.2%)	< 0.001
Vasopressor	2818 (58.4%)	327 (89.6%)	< 0.001	1219 (58.9%)	132 (88.6%)	< 0.001
Severity scales						
SAPSII	37 (30, 46)	54 (43, 64)	< 0.001	36 (29, 46)	52 (42, 63)	< 0.001
SOFA	5 (3, 7)	10 (8, 13)	< 0.001	4 (3, 7)	10 (6, 13)	< 0.001
Outcomes						
LOS in ICU	3.1 (1.8, 5.8)	11.5 (5.2, 20.9)	< 0.001	3.2 (2.0, 6.1)	11.2 (5.6, 23.9)	< 0.001
Hospital mortality, *n* (%)	872 (18.1%)	220 (60.3%)	< 0.001	358 (17.3%)	92 (61.7%)	< 0.001

Values were shown as median (interquartile range) unless otherwise indicated

*AKI* acute kidney injury, *BUN* blood urea nitrogen, *DM* diabetes mellitus, *eGFR* estimated glomerular filtration rate, *INR* international normalized ratio, *LOS* length of stay, *MAP* mean arterial pressure, *MV* mechanical ventilation, *pO*_*2*_ partial pressure of oxygen, *pCO*_*2*_ partial pressure of carbon dioxide, *RRT* renal replacement therapy, *SAPSII* simplified acute physiology score II, *SOFA* sequential organ failure assessment, *WBC* white blood cell

There were also significant differences in vital signs at AKI detection between RRT group and non-RRT group in both cohorts, such as MAP, respiratory rate, and heart rate. Patients with congestive heart failure and live cirrhosis were more likely to receive RRT. Vasopressors use and MV were more common in the RRT group. In terms of SAPSII and SOFA, patients in the RRT group were more critically ill than those in the non-RRT group. By comparison, patients who required RRT had significantly higher hospital mortality and longer LOS in ICU.

### Univariable and multivariable analyses of risk factors associated with RRT among AKI patients in ICU

The univariate logistic regression analysis of training cohort demonstrated that risk factors with statistical difference in the baseline comparisons were associated with the likelihood of RRT among AKI patients in ICU. Multivariate logistic regression analysis based on AIC identified that age, hemoglobin, sCr, BUN and lactate at AKI detection, comorbidity of congestive heart failure, AKI stage, vasopressor use, and SOFA score were independent risk factors for RRT in the training cohort. Besides, VIF was significantly < 10 indicating no collinearity among the independent variables (Table 2). The regression equation to predict the probability of RRT by multivariable logistic analysis is demonstrated in Additional file 1: Figure S1.

**Table 2:** Univariate and multivariate logistic regression analyses of risk factors of RRT in the training cohort

	Univariate (OR, 95% CI)	*P* value	Multivariate (OR, 95% CI)	*P* value	VIF
Age	0.968 (0.961, 0.974)	< 0.001	0.985 (0.972, 0.997)	0.015	1.1
Gender					
Male	1.0 (reference)		**–**	**–**	
Female	0.974 (0.782,1.213)	0.814	**–**	**–**	
Baseline eGFR	0.978 (0.975, 0.981)	< 0.001	–	–	
Laboratory results at AKI detection					
WBC count	1.034 (1.020, 1.048)	< 0.001	**–**	**–**	
Hemoglobin	0.887 (0.842, 0.934)	< 0.001	0.910 (0.845, 0.979)	0.011	1.1
Platelet	0.995 (0.993, 0.996)	< 0.001	**–**	**–**	
INR	1.768 (1.628, 1.920)	< 0.001	–	–	
sCr	1.733 (1.651, 1.820)	< 0.001	1.330 (1.187, 1.489)	< 0.001	2.2
BUN	1.039 (1.035, 1.043)	< 0.001	1.025 (1.017, 1.032)	< 0.001	1.9
pH	0.006 (0.003, 0.015)	< 0.001	**–**	**–**	
* p*O_2_	0.997 (0.996, 0.998)	< 0.001	**–**	**–**	
* p*CO_2_	0.982 (0.972, 0.993)	0.002	**–**	**–**	
Bicarbonate	0.835 (0.817, 0.854)	< 0.001	**–**	**–**	
Anion gap	1.206 (1.184, 1.228)	< 0.001	**–**	**–**	
Lactate	1.265 (1.236, 1.294)	< 0.001	1.158 (1.117, 1.200)	< 0.001	1.3
Sodium	0.933 (0.913, 0.954)	< 0.001	**–**	**–**	
Potassium	1.351 (1.217, 1.500)	< 0.001	**–**	**–**	
Chloride	0.964 (0.948, 0.981)	< 0.001	**–**	**–**	
Phosphate	1.642 (1.533, 1.759)	< 0.001	**–**	**–**	
Vital signs at AKI detection					
Temperature	1.189 (0.601, 1.888)	0.145	**–**	**–**	
MAP	0.957 (0.946, 0.969)	< 0.001	**–**	**–**	
Heart rate	1.020 (1.013, 1.028)	< 0.001	**–**	**–**	
Respiratory rate	1.132 (1.107, 1.157)	< 0.001	**–**	**–**	
Comorbidities					
Hypertension	0.386 (0.306, 0.487)	< 0.001	–	–	
DM	1.137 (0.885, 1.461)	0.315	**–**	**–**	
Congestive heart failure	1.373 (1.085, 1.739)	0.008	1.646 (1.203, 2.252)	0.002	1.1
Myocardial infarction	0.757 (0.509, 1.124)	0.167	**–**	**–**	
Liver cirrhosis	4.764 (3.626, 6.258)	< 0.001	**–**	**–**	
AKI stage					1.4
Stage 1	1.0 (reference)		1.0 (reference)		
Stage 2	3.303 (2.377, 4.589)	< 0.001	3.420 (2.288, 5.112)	< 0.001	
Stage 3	17.234 (13.200, 22.799)	< 0.001	5.455 (3.740, 7.956)	< 0.001	
Treatment					
MV	1.406 (1.108, 1.784)	0.005	**–**	**–**	
Vasopressor	6.141 (4.368, 8.634)	< 0.001	4.178 (2.690, 6.488)	< 0.001	1.3
Severity scales					
SAPSII	1.060 (1.053, 1.067)	< 0.001	**–**	**–**	
SOFA	1.393 (1.352, 1.435)	< 0.001	1.194 (1.17, 1.275)	< 0.001	1.8

*AKI* acute kidney injury, *BUN* blood urea nitrogen, *DM* diabetes mellitus, *eGFR* estimated glomerular filtration rate, *INR* international normalized ratio *LOS* length of stay, *MAP* mean arterial pressure, *MV* mechanical ventilation, *PO*_*2*_ partial pressure of oxygen, *PCO*_*2*_ partial pressure of carbon dioxide, *RRT* renal replacement therapy, *SOFA* sequential organ failure assessment, *SAPSII* simplified acute physiology score II, *VIF* variance inflation factor *WBC* white blood cell

### Construction and internal validation of the RRT predictive nomogram

**Fig. 2:**
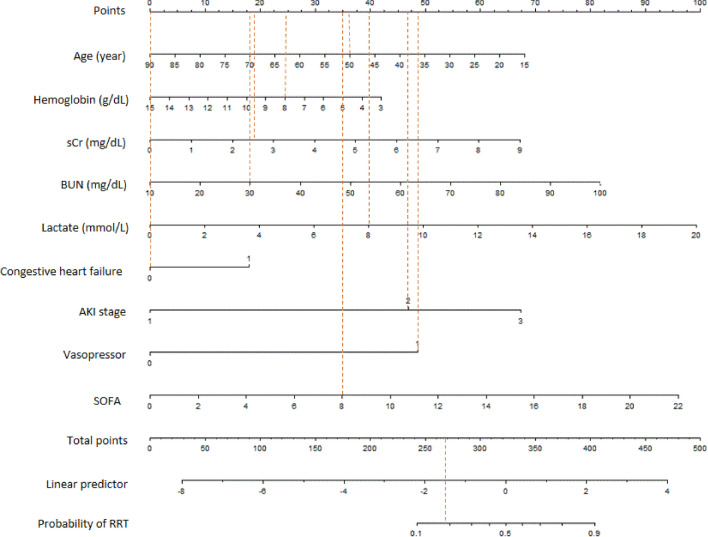
The nomogram prediction score of RRT among AKI patients in ICU. When using it, drawing a vertical line from each variable to the point axis for the score, then the points for all the parameters were added; finally, a line from the total point axis was drawn to correspond the probability of RRT at the bottom. *AKI* acute kidney injury, *BUN* blood urea nitrogen, *RRT* renal replacement therapy, *sCr* serum creatinine, *SOFA* Sequential Organ Failure Assessment

The nomogram for predicting the probability of RRT among AKI patients in ICU was constructed based on the multivariate logistic regression model (Fig. 2). Every specific value of these factors was allocated a score on the points scale. By adding up these scores, the total score was calculated. For example, a 50-year-old white male developed AKI stage 2 after admission to ICU. Laboratory findings at AKI detection were as follows: sCr 2.5 mg/dL, BUN 30 mg/dL, hemoglobin 8 g/dL, and lactate 8 mmol/L. He had no comorbidity of congestive heart failure and was treated with vasopressor. His SOFA score was 8. According to the nomogram, the results were as follows: age 50 = 36, sCr 2.5 = 19, BUN 30 = 18, hemoglobin 8 = 25, lactate 8 = 40, congestive heart failure 0 = 0, AKI stage 2 = 47, vasopressor 1 = 49, and SOFA 8 = 35. Therefore, the sum of above scores was 269, which indicated that the probability of RRT was around 20%.

**Fig. 3:**
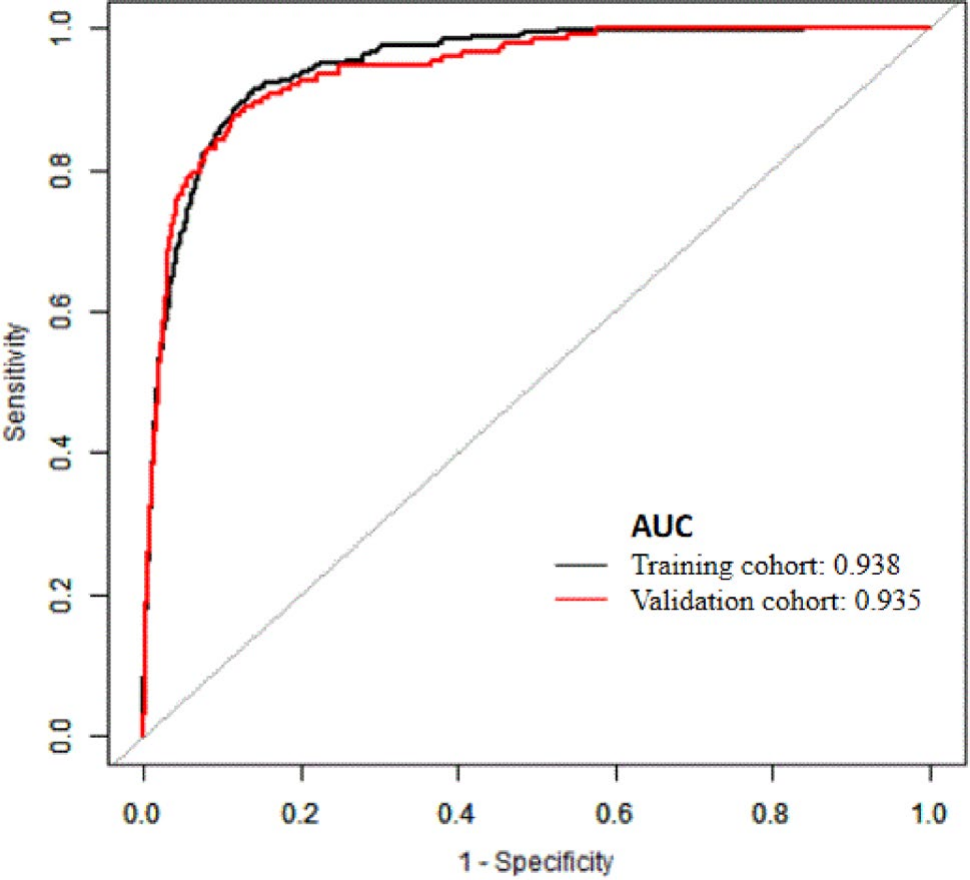
The receiver-operating characteristic (ROC) curves of the nomogram in the training cohort and validation cohort

**Fig. 4:**
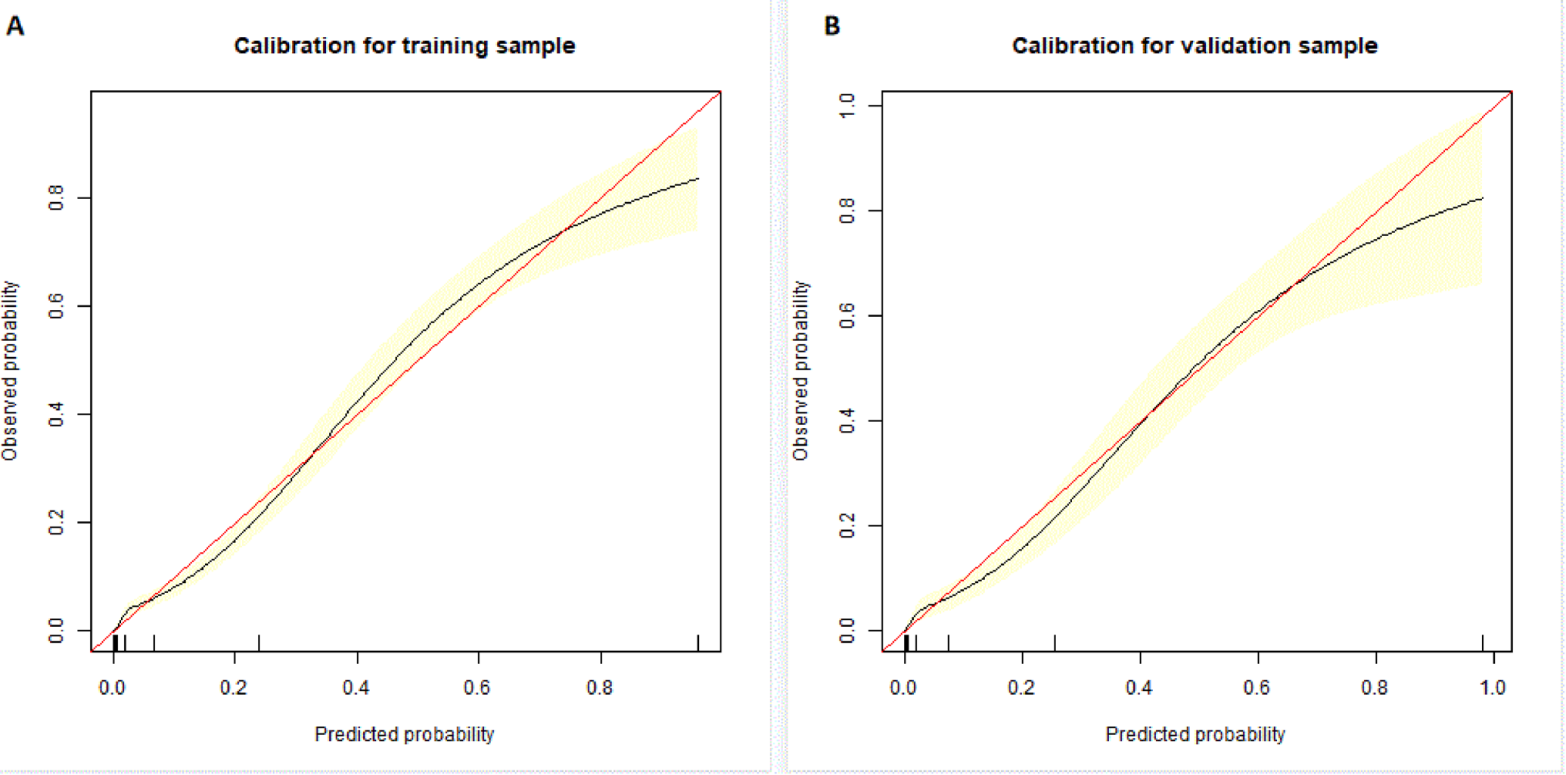
Calibration curve analysis in the training (**A**) and validation (**B**) cohorts. The horizontal axis represents the nomogram-predicted probability of RRT, and the vertical axis represents the actual observed probability of RRT. *RRT* renal replacement therapy

The discrimination power of the nomogram was evaluated by the *C*-index value and ROC curve (Fig. 3). The *C*-index values for the prediction of RRT were 0.938 (95% CI 0.927–0.949) in the training cohort with sensitivity 90.1% and specificity 85.2% and 0.935 (95% CI 0.919–0.951) in the validation cohort with sensitivity 85.9% and specificity 89.0%. In addition, the Hosmer–Lemeshow test yielded non-significant statistics in both training (*P* = 0.430) and validation cohorts (*P* = 0.392), which indicated that the calibration curves of the nomogram showed satisfactory concordance between the predictive and actual outcomes (Fig. 4). These results suggested that the nomogram had powerful predictive ability of RRT among AKI patients in ICU.

### Clinical use of the nomogram

**Fig. 5:**
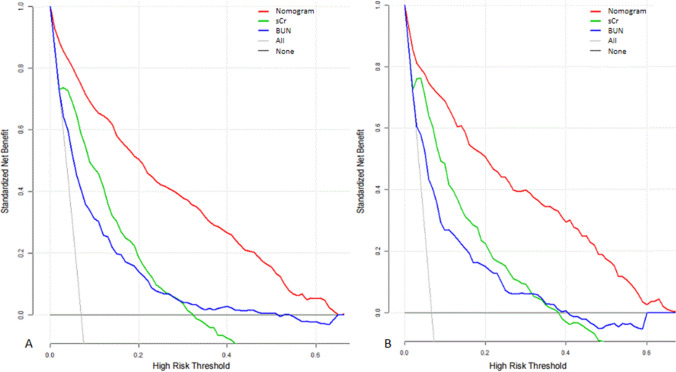
Decision curve analysis of the nomogram, Scr, and BUN for the risk of RRT in the training cohort (**A**) and validation cohort (**B**). *X*-axis indicates the threshold probability for RRT and *Y*-axis indicates the net benefit. The gray line represents the assumption that all patients are treated with RRT. The thin black link represents the assumption that no patients are treated with RRT. The net benefit was calculated by subtracting the proportion of all patients who are false positive from the proportion who are true positive, weighting by the relative harm of forgoing treatment compared with the negative consequences of an unnecessary treatment. Here, the relative harm was calculated by ($\frac{P\mathrm{t}}{1-P\mathrm{t}}$). *BUN* blood urea nitrogen, *P*_t_ threshold probability, *RRT* renal replacement therapy, *sCr* serum creatinine

The DCA curve was performed to determine clinical practice of this nomogram by quantifying the net benefits at different threshold probabilities. (Net benefit is defined as the proportion of true positives minus the proportion of false positives, weighted by the relative harm of false-positive and false-negative results.) The decision curve showed that if the threshold probability of a patient or doctor is 10–60%, using the nomogram to predict RRT adds more benefit than either the treat-all-patients scheme or the treat-none scheme and produced greater net benefit than sCr, and BUN (Fig. 5).

## Discussion

The application of RRT is mainly to treat AKI patients with refractory complications, such as hyperkalemia, acidemia, fluid overload, and toxicity. However, the frequencies of these urgent indications for RRT in critically care setting are less commonly occurred and could be avoided in most of time [13]. In ICU, the administration of RRT is usually to solve the problems of non-kidney organ dysfunction (e.g., lung and heart) and assist in mitigating other organ support, such as invasive mechanical ventilation. Due to the reason that there remains no reliable or commonly accepted tools to evaluate this kidney ‘demand supply’ relationship, it brings huge challenge for clinicians to make a decision of RRT for critically ill patients with AKI in a timely manner [14, 15].

In this study, we presented a precise nomogram based on abundant clinical information from the MIMIC-III database to predict the probability of RRT among AKI patients in ICU. Most of our data were measured at AKI detection, which allows timely intervention as compared with other large prospective studies that predicted adverse outcomes in patients already receiving RRT at baseline [16, 17]. Nine risk factors, including age, hemoglobin, sCr, BUN and lactate at AKI detection, comorbidity of congestive heart failure, AKI stage, SOFA score, and vasopressor use, were included to establish a predictive model. The good discrimination and calibration of the nomogram were demonstrated in both the training and validation cohorts. Thus, this nomogram could be efficiently and effectively applied in clinical practice to perform an individualized prediction of RRT treatment. As shown in Fig. 2, the scores of 9 variables can be easily acquired with vertical lines to the point axis. After calculating the total score, the probability of patients to be treated with RRT was demonstrated at the bottom. Then, decision curve analysis was further applied to see whether the nomogram-based decision could improve clinical outcomes. This novel method investigates clinical consequence by calculating net benefit at different threshold probabilities. Therefore, if the threshold probability of a patient is 20%, the net benefit would be 50% when using nomogram to initiate RRT. It can be stated that if we perform RRT based on the prediction model, compared to treat-none scheme, net benefit is equivalent to performing RRT in 50 AKI patients per 100 and treating no AKI patients who do not require RRT. Besides, using the threshold of the nomogram could produce more net benefit than sCr and BUN. The levels of sCr and BUN were used as cutoffs to define early and late initiation of dialysis in the previous studies [18, 19]. Although sCr and BUN could not fully reflect renal function, they retained their position among routinely ordered tests from most clinical chemistry laboratories.

Serum creatinine and blood urea nitrogen at AKI detection were still the leading causes to influence the decision of RRT. However, only relying on sCr, BUN or AKI stage did not meet the decision criteria of starting RRT according to the predictive nomogram. In one multicenter randomized trial, Gaudry et al. enrolled 630 patients who were admitted to the ICU with KDIGO Stage 3 AKI [5]. They found that there were no significant differences between early strategy of RRT (started immediately after randomization) and late strategy. The STARRT-AKI trial [20] compared early and late RRT initiation in 3019 critically ill patients with AKI (KDIGO Stage 2) without urgent indications. The results also demonstrated that preemptive RRT therapy did not bring any clinical benefits. The IDEAL-ICU study [21] concluded the same result that accelerating the initiation of RRT did not produce any clinical benefit in the absence of emergency indications.

As regard to hemoglobin concentration, it is an important component of arterial oxygen content, which plays a major role in oxygen delivery. Therefore, low hemoglobin concentration can exacerbate oxygen delivery to the kidney and result in tissue ischemia in the renal medulla [22]. Heart function is another important factor affecting renal oxygen delivery. The interplay between cardiac and renal dysfunctions has been recognized as a clinical entity dubbed cardiorenal syndrome (CRS) [23]. The fall in renal blood flow due to low cardiac output explains renal dysfunction caused by congestive heart failure [24].

Serum lactate is also a well-known biomarker to reflect the severity of circulatory failure and AKI. It has also been shown to the need for RRT in septic patients [25]. Our results also added important information about the predictive relevance of hyperlactatemia in AKI patients requiring RRT. Furthermore, higher SOFA score was also associated with RRT demand, which was consistent with the previous results [26, 27]. The use of vasopressors indicated severe hemodynamic instability in those critically ill. Recent data suggested that dobutamine may not improve microcirculatory perfusion in septic shock despite an increase in cardiac index [28]. Besides, some inotrope, such as adrenaline, aggravated perioperative tubular injury and leaded to a decrease in glomerular filtration rate [29]. It would be of interest to find the amount and category of vasopressors to predict RRT in critically ill patients with AKI. Last but not least, we found that increasing age was associated with a lower risk of AKI requiring RRT. A reason for this may be that elder critically ill patients were less likely to receive RRT than younger patients.

Several limitations should be addressed when interpreting the results of this study. First, we only included patients in a public database from a single center, which may limit generalizability of the results. Therefore, the model needs to be verified by multiple medical centers. Second, we used data from MIMIC-III database, raising the possibility of selection bias. Third, the etiology of AKI was also an important factor to determine whether to initiate RRT. However, due to the nature of retrospective study, it was difficult to find out the definite reason of AKI, which prevented us from discussing this aspect. Finally, our nomogram was limited by the retrospective nature of data collection and failed to evaluate the survival benefit of RRT initiation based on nomogram strategy. Further efforts on prospective data collection and clinical application are encouraged to verify the model.

## Conclusion

In summary, the nomogram, composed of age, hemoglobin, sCr, BUN and lactate at AKI detection, comorbidity of congestive heart failure, AKI stage, and vasopressor use, demonstrated high accuracy in predicting the risk of RRT at the time of AKI diagnosis in ICU. This predictive tool may help clinicians to deliver proper care to AKI patients in ICU and bring benefit to indicate AKI patients who would not need RRT.

### Supplementary Information

Below is the link to the electronic supplementary material.

Supplementary file1 (DOCX 41 KB)

## Data Availability

The data generated and analyzed during the current study are available on the MIMIC-III website at https://physionet.org/content/mimiciii/1.4
