# Supplementary material for: Development and validation of a nomogram to predict the risk of renal replacement therapy among acute kidney injury patients in intensive care unit
Source: Clin Exp Nephrol. 2023 Jul 27;27(11):951–60. doi: 10.1007/s10157-023-02383-5 (PMC10581925; doi:10.1007/s10157-023-02383-5)
Supplement: Supplementary file 1 — Supplementary file1 (DOCX 41 KB) [file 10157_2023_2383_MOESM1_ESM.docx]

Table S1. Missing number for risk variables and outcome variables

| **Risk Variables** | Missing number (%) | **Risk Variables** | Missing number (%) |
| --- | --- | --- | --- |
| Age | 0 (0) | Chloride | 65 (0.4) |
| Gender | 0 (0) | Phosphate | 403 (2.3) |
| WBC | 101 (0.6) | Temperature | 560 (3.2) |
| Hemoglobin | 50 (0.3) | MAP | 156 (0.9) |
| Platelet | 63 (0.4) | Heart rate | 156 (0.9) |
| INR | 1194 (6.9) | Respiratory rate | 171 (1.0) |
| sCr | 0 (0) | Hypertension | 0 (0) |
| BUN | 30 (0.2) | DM | 0 (0) |
| pH | 3443 (19.8) | Heart failure | 0 (0) |
| PO_2_ | 3443 (19.8) | Myocardial infarction | 0 (0) |
| PCO_2_ | 3443 (19.8) | Liver cirrhosis | 0 (0) |
| Bicarbonate | 92 (0.5) | AKI stage | 0 (0) |
| Anion gap | 431 (2.5) | Vasopressor | 0 (0) |
| Lactate | 3329 (19.1) | Mechanical ventilation | 0 (0) |
| Sodium | 25 (0.1) | SOFA | 0 (0) |
| Potassium | 19 (0.1) | SAPSII | 0 (0) |

*AKI, acute kidney injury; BUN, blood urea nitrogen; DM, diabetes mellitus; eGFR, estimated glomerular filtration rate; INR, international normalized ratio; LOS, length of stay; MAP, mean arterial pressure; MV, mechanical ventilation; PO_2_, partial pressure of oxygen; PCO_2_, partial pressure of carbon dioxide; RRT, renal replacement therapy; sCr, serum creatinine; SOFA, Sequential Organ Failure Assessment; SAPSII, Simplified Acute Physiology Score II; WBC, white blood cell.*

Figure S1. The equation to predict the probability of RRT by multivariate logistic regression analysis.


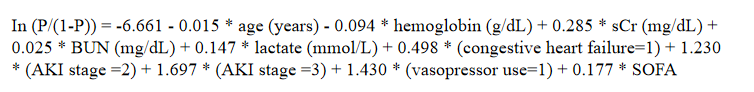


*AKI, acute kidney injury; BUN, blood urea nitrogen; sCr, serum creatinine; RRT, renal replacement therapy; SOFA, Sequential Organ Failure Assessment.*
